# Autophagy-Related Genes Are Involved in the Progression and Prognosis of Asthma and Regulate the Immune Microenvironment

**DOI:** 10.3389/fimmu.2022.897835

**Published:** 2022-05-10

**Authors:** Fan Yang, Jingwei Kong, Yuhan Zong, Zhuqing Li, Mingsheng Lyu, Wanyang Li, Wenle Li, Haoyue Zhu, Shunqi Chen, Xiaoshan Zhao, Ji Wang

**Affiliations:** ^1^ College of Traditional Chinese Medicine, Beijing University of Chinese Medicine, Beijing, China; ^2^ National Institute of Traditional Chinese Medicine (TCM) Constitution and Preventive Medicine, Beijing University of Chinese Medicine, Beijing, China; ^3^ Center of Respiratory, Beijing University of Chinese Medicine Affiliated Dongzhimen Hospital, Beijing, China; ^4^ Department of Respiratory, The Third Affiliated Hospital, Beijing University of Chinese Medicine, Beijing, China; ^5^ Department of Clinical Nutrition, Chinese Academy of Medical Sciences - Peking Union Medical College, Peking Union Medical College Hospital, Beijing, China; ^6^ Beijing Hospital of Traditional Chinese Medicine (TCM), Capital Medical University, Beijing, China; ^7^ School of Chinese Medicine, Southern Medical University, Guangzhou, China

**Keywords:** autophagy-related genes, asthma, immune cell, prognosis, diagnostic model

## Abstract

**Background:**

Autophagy has been proven to play an important role in the pathogenesis of asthma and the regulation of the airway epithelial immune microenvironment. However, a systematic analysis of the clinical importance of autophagy-related genes (ARGs) regulating the immune microenvironment in patients with asthma remains lacking.

**Methods:**

Clustering based on the k-means unsupervised clustering method was performed to identify autophagy-related subtypes in asthma. ARG-related diagnostic markers in low-autophagy subtypes were screened, the infiltration of immune cells in the airway epithelium was evaluated by the CIBERSORT, and the correlation between diagnostic markers and infiltrating immune cells was analyzed. On the basis of the expression of ARGs and combined with asthma control, a risk prediction model was established and verified by experiments.

**Results:**

A total of 66 differentially expressed ARGs and 2 subtypes were identified between mild to moderate and severe asthma. Significant differences were observed in asthma control and FEV1 reversibility between the two subtypes, and the low-autophagy subtype was closely associated with severe asthma, energy metabolism, and hormone metabolism. The autophagy gene *SERPINB10* was identified as a diagnostic marker and was related to the infiltration of immune cells, such as activated mast cells and neutrophils. Combined with asthma control, a risk prediction model was constructed, the expression of five risk genes was supported by animal experiments, was established for ARGs related to the prediction model.

**Conclusion:**

Autophagy plays a crucial role in the diversity and complexity of the asthma immune microenvironment and has clinical value in treatment response and prognosis.

## Introduction

Asthma is a chronic airway inflammatory disease involving a variety of cells and cellular components. At least 300 million patients have asthma worldwide, and the incidence is increasing yearly (1). Current studies showed that the Th1/Th2-mediated immune imbalance is the main mechanism of asthmatic airway inflammatory response (2), and a variety of immune cells are involved in the course of asthma. In addition, the Global Initiative for Asthma (2021) updated the definition of severe asthma. This group accounts for about 10% of the total number of patients but accounts for 60% of the expenditure on asthma medicine (3–5). Therefore, effective assessment tools are urgently needed for the early identification and management of this population to prevent the transition from mild to moderate asthma to severe asthma and develop drugs for severe, uncontrolled asthma (6).

Autophagy, also known as cell self-digestion, is a process in which the body relies on lysosomes to degrade excessive proteins and aging and damaged organelles for the recycling of body substances and maintenance of the balance of cell metabolism and homeostasis. A growing number of studies showed a close relationship between autophagy and asthma (7). For example, autophagy is a cellular mechanism of TGFβ1-dependent airway remodeling and loss of lung function in patients with asthma, and the single nucleotide polymorphism rs12212740 of autophagy-related gene 5 (ATG5) is significantly associated with lung function and airway remodeling in patients with asthma (8). In addition, in asthma, the regulation of immune cells by autophagy is extensive (9). Clinical studies showed that the autophagy marker LC3-II is highly expressed in neutrophils, T lymphocytes in sputum and neutrophils, and eosinophils and monocytes in the peripheral blood of patients with asthma, and the expression levels of autophagy pathway-related proteins Beclin-1, LC3B, and ATG5 are significantly increased in lung eosinophils compared with the control group (10–12). The dendritic cell-specific knockout of ATG5 can reduce the expression of cytokines (e.g., IL-6, TNF, and IL-1β) and aggravate the pulmonary inflammatory response in bronchial asthma mouse models (13). Although current studies preliminarily reported the role of autophagy and its related genes in the occurrence and development of asthma, most studies focused on specific ARGs and did not comprehensively analyze ARGs in combination with the clinical prognosis and immune imbalance of asthma. In order to clarify the relationship among autophagy, immune cells and asthma, and how these biological processes affect the progression and prognosis of asthma, in this study, based on a group of transcriptome data sets of airway epithelium of patients with asthma, we conducted a comprehensive analysis of ARGs to explore the diagnostic and prognostic value of ARGs in patients with asthma, and the accuracy of the diagnostic model was further verified by an asthma animal model.

## Materials and Methods

### Data Source

We downloaded the asthma microarray data set GSE89809, including airway epithelial samples from 27 patients with mild to moderate asthma and 11 patients with severe asthma (14), from the GEO (https://www.ncbi.nlm.nih.gov/geo/) database from GPL13158 (Affymetrix HT HG-U133+ PM Array Plate). Raw data were background corrected and normalized by the RMA algorithm. Differentially expressed genes (DEGs) were screened using the “*limma*” software package (15), and *P* < 0.05 and |log2FC| > 0.585 were considered to indicate significant differences. ARGs were extracted from the Human Autophagy Database (http://www.autophagy.lu/index.html) and the GO_AUTOPHAGY gene set in the Gene Set Enrichment Analysis (GSEA) website (http://software.broadinstitute.org/gsea/index.jsp). In accordance with Ref., these two gene sets were merged into one ARG set (16).

### Classification and Functional Enrichment Analysis of ARG-Related Subtypes in Asthma

The unsupervised cluster analysis was performed to identify different subtypes on the basis of the differences in ARG expression in asthma (17, 18). A consensus clustering algorithm was used to evaluate cluster numbers and robustness. The R package “*ConsensuClusterPlus*” implemented the above steps for 1000 iterations to guarantee the robustness of classification (19). The protein–protein interaction (PPI) network was obtained from the STRING database (https://string-db.org/), and the MCODE was performed using the Metascape (http://metascape.org/gp/index.html#/main/step1) platform. We used the “*clusterProfiler*” and “*ggplot2*” packages to perform Gene Ontology (GO) and Kyoto Encyclopedia of Genes and Genomes (KEGG) enrichment analyses, respectively, on DEGs (20, 21). GSEA was performed on the gene expression matrix through the “*clusterProfiler*” package, and the “*c2.cp.kegg.v7.0.symbols.gmt*” was selected as the reference gene set. Genes associated with specific subtypes were identified by the weighted gene co-expression network analysis (WGCNA) by using the R package “*WGCNA*” (22). The topological overlap matrix (TOM) and the corresponding dissimilarity (1 − TOM) were transformed from the adjacency matrix. A hierarchical clustering dendrogram was further built, and similar gene expressions were divided into different modules. Finally, the expression profiles of each module were summarized by the module eigengene (ME), and the correlation between the ME and clinical features was determined.

### Relationship Between the Screening and Validation of ARG-Related Diagnostic Markers and Immune Cell Infiltration

We used the least absolute shrinkage and selection operator (LASSO) logistic regression and the support vector machine–recursive feature elimination (SVM-RFE) to perform feature selection and screen diagnostic markers for asthma (23, 24). The LASSO algorithm was applied with the “*glmnet*” package (25). The SVM module was established to further identify the diagnostic value of these biomarkers in asthma by the “*e1071”* package (26). Subsequently, the degree of immune cell infiltration in the airway epithelium was assessed. The CIBERSORT algorithm is an excellent tool used to calculate the abundance of specific cells in the mixture matrix (27). The “*Corrplot*” software package was further used to draw relevant heatmaps and show the correlation of 22 types of infiltrating immune cells (28), and the differences in infiltrating immune cells between groups were illustrated by the “*ggplot2*” package violin plot. Finally, the Spearman correlation analysis of diagnostic markers and infiltrating immune cells was performed using the “*ggstatsplot*” package, and results were visualized using the “*ggplot2*” package (29).

### Construction of a Risk Signature Associated With Asthma Control

Univariate Cox regression models were used to select genes associated with asthma control. *P* < 0.05 was considered statistically significant. The LASSO regression was used to screen out the optimal gene combination and construct the risk characteristics, and the regression coefficient was linearly combined with the gene expression level to establish the risk characteristics. The risk score was calculated as follows: Risk score = (exprgene1 × Coefgene1) + (exprgene2 × Coefgene2) + … + (exprgene*n* × Coefgene*n*) (30). Patients with asthma were divided into low- and high-risk groups in accordance with the median risk score. The Kruskal test was used to compare the infiltrating immune cell abundance score and immune response score in the two risk models.

### Gene Expression Regulation of Prognostic Genes, Autophagy-Related Diagnostic Markers, and Predictive Models

The transcriptional regulatory networks of prognosis-related genes were predicted from the ChEA3 database (https://maayanlab.cloud/chea3/). The database integrated ENCODE; ReMap; some independently published CHIP-seq data; and transcription factor co-expression data from GTEx, TCGA, and ARCHS4 RNA-seq data (31). The target miRNA was first predicted using the starBase (http://starbase.sysu.edu.cn/) database to predict the regulation of target genes by noncoding RNAs, and prediction results included the analysis of RNA22, miRanda, and TargetScan (32). Then, the target lncRNA of miRNA was predicted using the miRNet2.0 database (www.mirnet.ca/miRNet/home.xhtml) and starBase. Finally, the ceRNA network was established (33, 34).

### Identification of Small Molecular Therapeutic Agents

The Broad Institutes Connectivity Map (cMAP) database (https://portals.broadinstitute.org/cmap) was used for the identification of small candidate molecules related to asthma (35). For the identification of small candidate chemical molecules, DEGs (log2FC > 0.585) were introduced into the cMAP database for GSEA. The PubChem (https://pubchem.ncbi.nml.gov) was used for the extraction of detailed information and 3D confirmation of the established small molecules. Then, molecular docking experiments were carried out, and the crystal structures of key targets were retrieved from the human (Human) database in the PDB (https://www.rcsb.org/) database. AutoDockTool software was used to dewater and hydrogenate the receptor protein, and Discovery Studio 4.5.0 software was used to perform molecular docking between small drug molecules and target proteins. A negative value indicates free binding. In this study, binding energy value less than − 5 kcal/mol (20.9 kj/mol) was set as significant binding.

### Animal Experiment

BALB/c mice (female, 17–20 g, 6–8 weeks old) were obtained from Beijing Vital River Laboratory Animal Technology Co., Ltd in China. All animal studies were conducted in accordance with the institutional animal care regulations of Beijing University of Chinese Medicine and were conducted in accordance with AAALAC and IACUC guidelines. Keeping mice in specific pathogen-free conditions in Beijing University of Chinese Medicine. All mice were kept at a controlled room (25 ± 1°C, 45–60% humidity). The allergic asthma model of mice was established by OVA sensitization and atomization inhalation stimulation. Briefly, mice were intraperitoneally injected with 2 mg of OVA (Sigma-Aldrich, Cat#A5503) mixed with 2 mg Imject™Alum Adjuvant (Invitrogen, Cat#77161) and PBS on day 0 and day 14. The challenge phase was from the 21st day to the 25th day after injection, and the mice were atomized with 1% OVA for 30 minutes. Then, the animals were killed, and part of the lung tissue was taken and placed in 4% paraformaldehyde fixed solution for the preparation of histopathological sections. The remaining lung tissues were washed with PBS and stored in - 80°C refrigerator for reverse transcription quantitative real-time polymerase chain reaction (RT - qPCR).

### Immunohistochemical Analysis

Rabbit anti-PTK6 polyclonal antibody (abs117941), rabbit anti-MAP2K7 polyclonal antibody (abs136429) and rabbit anti-CD46 polyclonal antibody (abs136051) were purchased from Aibixin Biotechnology Co., Ltd. (Shanghai, China). Horseradish peroxidase-labeled goat anti-rabbit IgG antibodies (GB23303) were purchased from Wuhan Servicebio Technology Co., Ltd. After being dewaxed, the lung slices were subjected to antigen recovery with citrate buffer under microwave heating. The slices were cooled down to room temperature and then sealed with 3% bovine serum albumin (BSA) for 30min and were incubated overnight with primary antibody at 4°C. The primary antibodies used were rabbit anti-PTK6 antibody (diluted 1:200), rabbit anti-MAP2K7 antibody (diluted 1:200), and rabbit anti-CD46 antibody (diluted 1:200). The slices were then washed with PBS and incubated with goat anti-rabbit secondary antibody (diluted 1:200) at 37°C for 50 min. After being rinsed with PBS, the slices were visualized with diaminobenzidine and counterstained with hematoxylin.

### Quantitative Real-Time Polymerase Chain Reaction

mRNA was extracted from the lung tissue using a universal RT-PCR Kit (Solarbio Science & Technology Co., Ltd., Shanghai, China) following the manufacturer’s instructions. Samples were treated with DNase and then purified using an RNeasy kit (Qiagen, Hilden, Germany). Glyceraldehyde-3-phosphate dehydrogenase (GAPDH) was used as internal reference. PCR primer sequences included the following: ACBD5: forward primer: 5’-TCGCAGGCGAAATTATCTTTG-3’; reverse primer: 5’-GTGCCAACCACTGAGCAATAA-3’, S100A9: forward primer: 5’-CATAAATGACATCATGGAGGACC-3’; reverse primer: 5’-TTGCCATCAGCATCATACACTC-3’, CD46: forward primer: 5’-TGGAAGGCAGTAGCATGGTGAT-3’; reverse primer: 5’-GAGGCTTGGTAGGATGAGTAGGC-3’, MAP2K7: forward primer: 5’-CAATGACTTGGAGAACTTGGGTG-3’; reverse primer: 5’-CGCCGCATTTGCTTAACAG-3’, PTK6: forward primer: 5’-CTGCGTGACTCTGATGAGAAAGC-3’; reverse primer: 5’-AGGTCACGGTGGATGTAATTCTG-3’, GAPDH: forward primer: 5’-CCTCGTCCCGTAGACAAAATG-3’; reverse primer: 5’-TGAGGTCAATGAAGGGGTCGT-3’.

### Statistical Analysis

Data were shown as mean ± standard deviation (SD). The differences between the two groups were evaluated by independent sample t-test and nonparametric test. A *P* < 0.05 was considered statistically significant. Statistical analyses and figures were obtained using IBM SPSS Statistics 23.0 (IBM SPSS Software, NY, USA) and GraphPad Prism Version 8.0 (GraphPad Software, San Diego, CA, USA).

## Results

### Landscape of ARGs Between Mild to Moderate and Severe Asthma Samples

**Figure 1:**
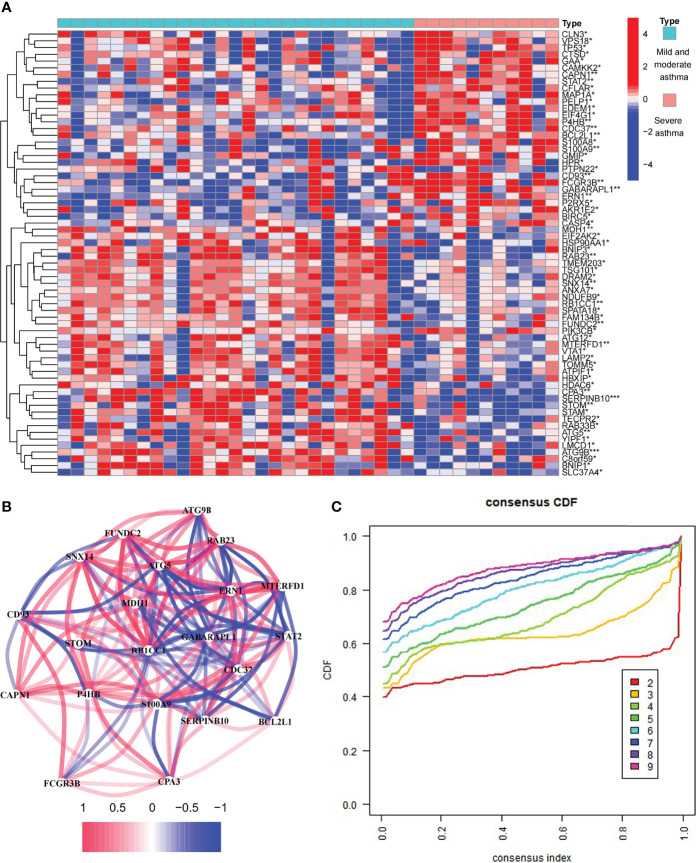
Expression of autophagy-related genes (ARGs) in asthma. **(A)** 66 differentially expressed ARGs between mild to moderate and severe asthma. *P < 0.05, **P < 0.01, ***P < 0.001. **(B)** Co-expression network of 22 ARGs. **(C)** Consensus clustering cumulative distribution function (CDF) for k = 2–9.

The study involved 531 ARGs. A total of 66 differentially expressed ARGs were observed in mild to moderate and severe asthma. Among these ARGs, SERPINB10 and ATG9B had the highest degree of difference (*P* < 0.001, **Figure 1A**). These ARGs had a PPI network (**Figure S1A**), and the MCODE analysis showed that this network had a key module (**Figure S1B**). Co-expression relationships were explored for 21 differentially expressed ARGs at *P* < 0.01, and a close correlation between genes was found (**Figure 1B**). These findings suggested that ARGs were involved in the disease process of asthma and might be related to the evolution of clinical symptoms or course of disease.

### Differentially Expressed ARGs Between Mild to Moderate and Severe Asthma Divided Asthma Into Two Subtypes

**Figure 2:**
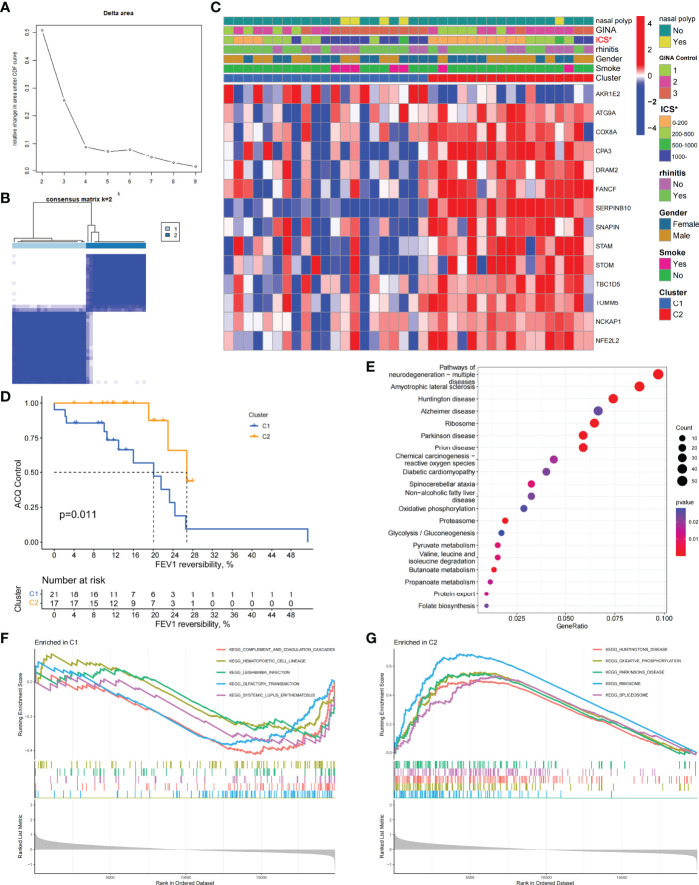
Two different autophagy-related subtypes identified in asthma by unsupervised clustering of 66 ARGs. **(A)** Relative change in area under CDF curve for k = 2–9. **(B)** Heatmap of the matrix of co-occurrence proportions for asthma samples. **(C)** Composite heatmap showing the relationship between the expression characteristics of 14 ARGs and the incidence of nasal polyps, GINA control, ICS dosage, prevalence of allergic rhinitis, gender, and smoking. *P < 0.05. **(D)** Kaplan–Meier curves of different gene subtypes ACQ Control and FEV1 reversibility. **(E)** KEGG analysis revealing the key signal pathway of C1 subtype. **(F, G)** GSEA of C1 and C2 subtypes.

To investigate the role of ARGs in asthma, we performed the unsupervised consensus cluster analysis on asthma samples based on the expression of 66 ARGs in combination with clinical data (**Figures 1C** and **2A, B**). Differences in the expression of 14 ARGs were observed in the two different asthma subtypes (C1 and C2), and the expression of ARGs was downregulated in C1-subtype samples, which were the low-autophagy subtype. The analysis of relevant clinical features showed a significant difference in the use of ICS between patients with C1 and C2 subtypes (*P* < 0.05). The C1 subtype was higher than the C2 subtype, suggesting the presence of glucocorticoid resistance, and patients with the C1 subtype had more severe symptoms than patients with the C2 subtype (**Figure 2C**). Other key clinical indicators also supported this result. The asthma control questionnaire (ACQ) is the most widely used standardized questionnaire for asthma control and can evaluate the control degree of patients stably and effectively (36–38). The FEV1 reversibility is a marker of airway hyper-responsiveness (AHR) and has traditionally been considered positively correlated with the severity of asthma (39, 40). A combined analysis of two key indicators, i.e., ACQ control and FEV1 reversibility, found that patients with the C1 subtype had higher FEV1 reversibility and more severe poor asthma control than those with the C2 subtype (**Figure 2D**). Even at the same level of FEV1 reversibility, patients with the C1 subtype had lower asthma control than those with the C2 subtype (*P* = 0.011). These results suggested that ARGs were involved in the disease exacerbation process of asthma, associated with clinical outcomes, and had a good classification function.

### Biological Characteristics of the C1 Subtype

**Figure 3:**
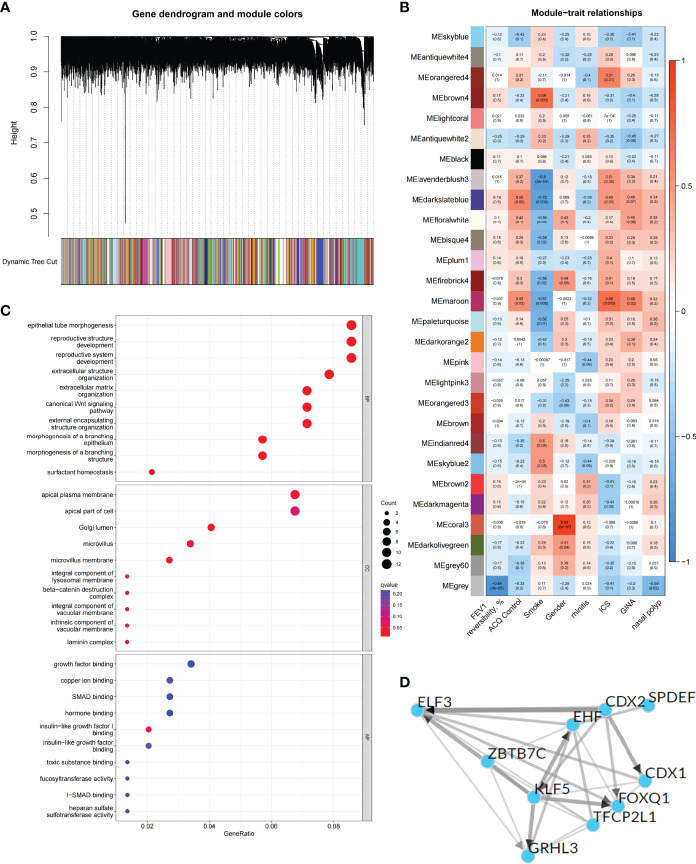
Identification and functional analysis of C1 subtype phenotype-related genes. **(A)** WGCNA of the C1 subtype to obtain a cluster dendrogram of coexpressed genes. **(B)** Module–trait relationships for C1 subtypes. Each module contains the corresponding correlation and *P*-value. **(C)** GO analysis of genes represented by the MEdarkslateblue module revealing biological processes associated with prognosis. **(D)** Transcription factors that regulate the expression of genes represented by the MEdarkslateblue module and their interactions.

To explore the biological characteristics of the C1 subtype, we performed GO analysis on the DEGs of C1 subtype. Results showed that the generation of precursor metabolites and energy, hormone metabolic process, and cilium assembly were the main biological processes; ribosomes and mitochondria were the key cell components; and oxidoreductase activity was the main molecular function (**Figure S1C**). The KEGG analysis revealed that neurodegeneration, amino acid metabolism, and energy metabolism were core pathways in this group (**Figure 2E**). GSEA showed the difference of main signal pathways between C1 and C2 subtypes (**Figures 2F, G**), in which the C1 subtype was predominantly enriched in complement and coagulation cascades, hematopoietic cell lineage, and other pathways. We further constructed the WGCNA network and explored biomarkers in C1-subtype samples on the basis of clinical features. Gene modules were detected on the basis of the TOM, and 28 modules were detected (**Figures 3A, B**). Further analysis of the relationship between modules and clinical features showed that MEdarkslateblue modules had the highest correlation with asthma control (COR = 0.58, *P* = 0.02). Therefore, the MEdarkslateblue module was selected for subsequent analysis. GO analysis showed that the characteristic genes in the module were involved in the Wnt signaling pathway, positive regulation of nitric oxide biosynthetic process, and extracellular matrix organization, which were closely related to AHR and airway remodeling in asthma (**Figure 3C**) (41). Genes within the same cluster often shared common transcription factors, and we predicted and analyzed the transcription factors of genes in the MEdarkslateblue module and visualized the mutual regulatory relationships between the top 10 transcription factors in Mean Rank (**Figure 3D**). The association of these transcription factors with asthma was demonstrated. For example, histone deacetylase 4 can mediate KLF5 deacetylation to upregulate CXCL12, leading to airway remodeling and promoting the progression of asthma (42).

### Correlation of Differentially Expressed ARGs Between Subtypes and Immune Cells

**Figure 4:**
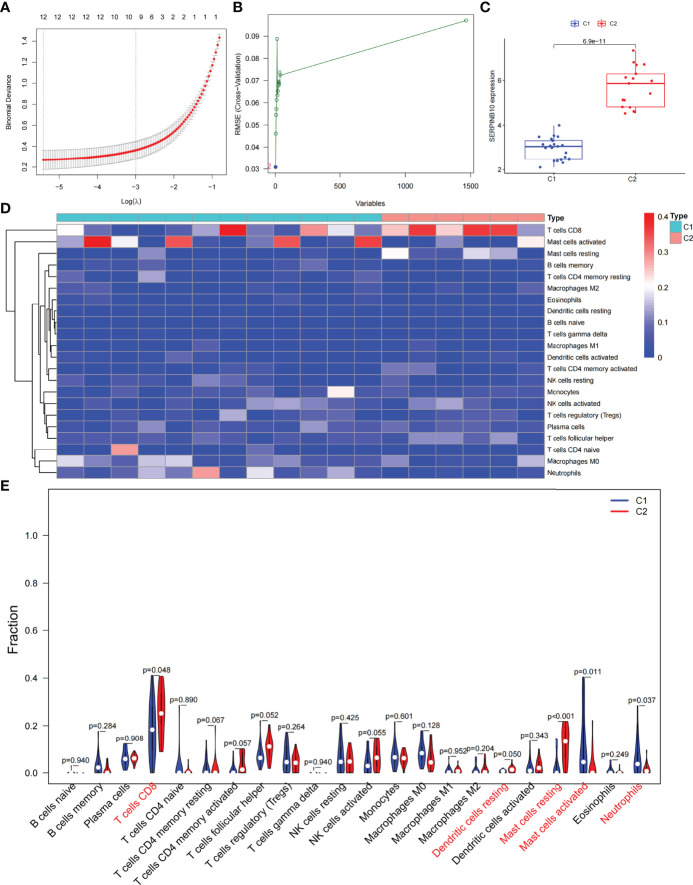
Screening of autophagy-related diagnostic markers and differences in immune cell infiltration between C1 and C2 subtypes. **(A)** LASSO logistic regression algorithm to screen diagnostic markers. **(B)** SVM-RFE algorithm to screen diagnostic markers. **(C)** Difference of SERPINB10 gene expression between C1 and C2 subtypes. **(D)** Heatmap of the degree of infiltration of 22 immune cells in C1 subtype samples versus C2 subtype samples. **(E)** Violin diagram of the proportion of 22 kinds of immune cells. Markers in red indicate significant differences between the two subtypes.

**Figure 5:**
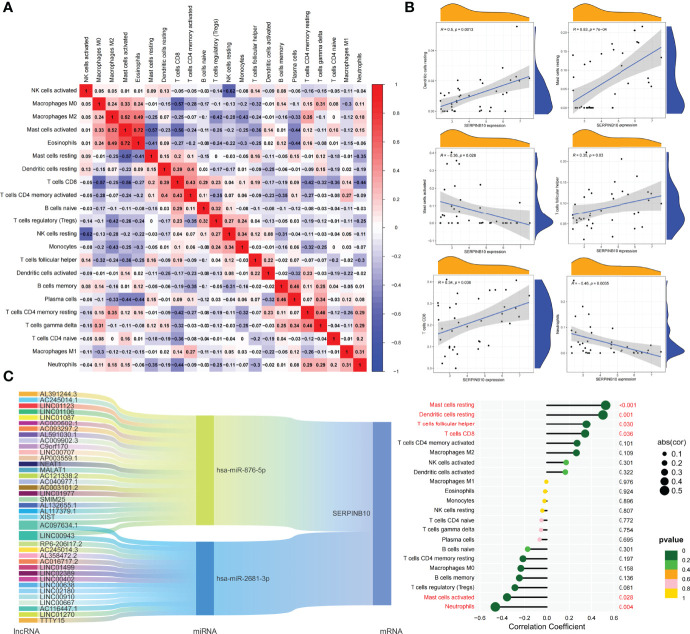
Correlation of key ARGs with immune cell infiltration and its gene expression regulatory network. **(A)** Heatmap of correlations of 22 immune cells. The size of the colored square represents the strength of correlation, and red and blue colors indicate positive and negative correlations, respectively. A dark color indicates a strong correlation. **(B)** Mountain diagram showing the correlation between the SERPINB10 gene and resting dendritic cells, resting mast cells, activated mast cells, T follicular helper cells, CD8^+^ T cells, and neutrophils. The lollipop diagram shows the correlation between SERPINB10 gene and 22 kinds of immune cells, and red marks indicate *P* < 0.05. **(C)** CeRNA networks involved in the regulation of SERPINB10 gene expression.

Based on the DEGs between C1 and C2 subtypes, we identified 12 genes as diagnostic markers for asthma by using the LASSO logistic regression algorithm (**Figure 4A**). Two genes were identified as diagnostic markers for asthma by using the SVM-RFE algorithm (**Figure 4B**). The genetic markers obtained by the two algorithms overlapped to obtain SERPINB10 as the diagnostic gene. The expression of SERPINB10, an ARG, in the C1 subtype was significantly lower than that in the C2 subtype (*P* < 0.01, **Figure 4C**). To assess the immune landscapes of C1 and C2 subtypes, we quantified the level of immune cell infiltration, and the ratios and differences of 22 types of immune cell infiltration between the two subtypes were presented as heatmaps and histograms (**Figures 4D** and **S1D**). The violin diagram of differences in immune cell infiltration showed that compared with the C2 subtype, the C1 subtype had less CD8^+^ T cell, resting dendritic cell, and resting mast cell infiltrations and more activated mast cell and neutrophil infiltrations (*P ≤* 0.05, **Figure 4E**). A strong correlation was also observed between several types of immune cells closely associated with asthma. CD8^+^ T cells was significantly negatively correlated with activated mast cells and M0 macrophages, whereas activated mast cells were significantly positively correlated with eosinophils and M2 macrophages (**Figure 5A**). The correlation analysis between SERPINB10 and immune cells showed that SERPINB10 was positively correlated with CD8^+^ T cells (R = 0.34, *P* = 0.036), T follicular helper cells (R = 0.35, *P* = 0.03), resting dendritic cells (R = 0.5, *P* = 0.0013), resting mast cells (R = 0.53, *P* = 7e−0) and negatively correlated with activated mast cells (R = −0.36, *P* = 0.028) and neutrophils (R = −0.46, *P* = 0.0035; **Figure 5B**). Finally, to explore the gene expression regulatory network of SERPINB10, a key ARG, we explored the ceRNA mechanism of SERPINB10 and constructed a ceRNA network including 38 lncRNAs and 2 miRNAs (**Figure 5C**). The ERPINB10 gene is regulated by miR-876-5p and miR-2681-3p, and the downregulation of miR-876-5p and miR-2681-3p may lead to the upregulation of SERPINB10 gene in asthma.

### Prognostic Model Classification, Immunological Characteristics, and Drug Prediction Based on Asthma Control

**Figure 6:**
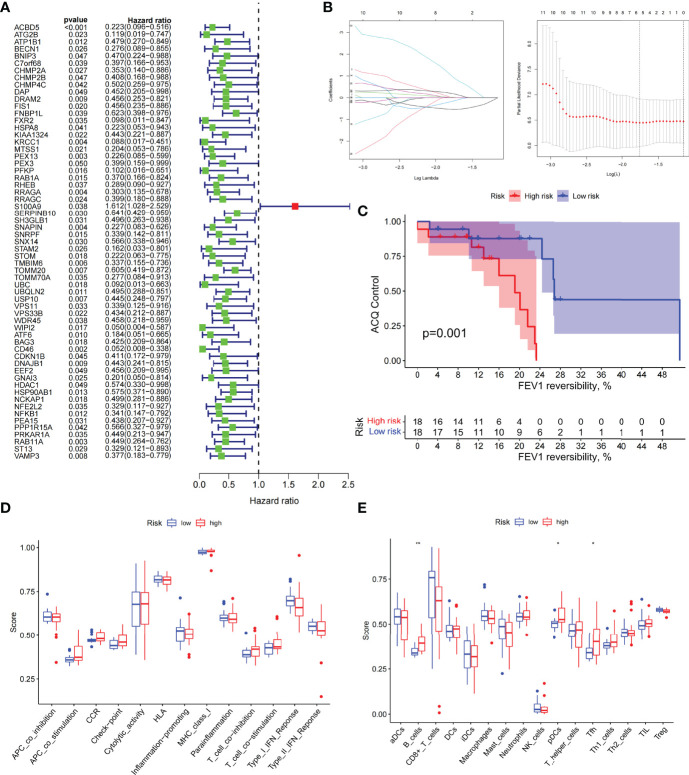
The prognostic model constructed by differentially expressed genes of different autophagy patterns can distinguish between high- and low-risk patients with asthma. **(A)** DEGs associated with asthma control status. Red and green colors represent high- and low-risk genes, respectively. **(B)** Distribution of LASSO coefficients for DEGs. Tenfold cross-validation for tuning parameter selection in the LASSO regression. Dotted vertical lines are drawn at the optimal values by minimum criteria and 1 − SE criteria. **(C)** Kaplan–Meier curves of ACQ control and FEV1 reversibility in patients with different risk groups. **(D)** Differences in the degree of response to each immune function in high- and low-risk groups. **(E)** Differences in the abundance of infiltrating immune cells in the immune microenvironment between high- and low-risk groups. *P < 0.05, **P < 0.01.

**Figure 7:**
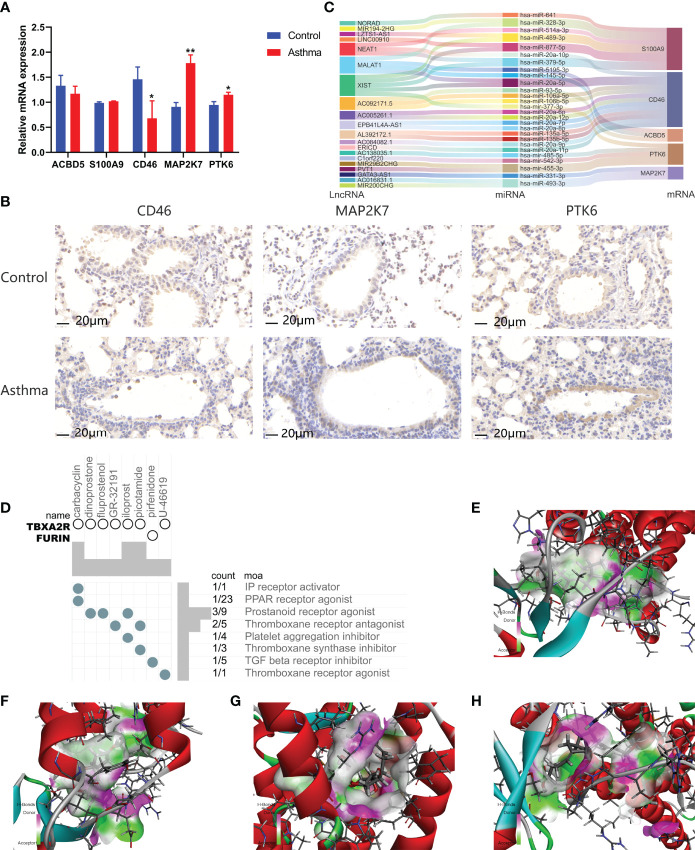
Gene expression regulatory network based on prognostic model and prediction of potential therapeutic drugs for high-risk asthma (N=3, *P < 0.05, **P < 0.01). **(A)** mRNA expression of 5 risk genes in lung tissues of asthmatic mice and normal mice. **(B)** The expression of CD46, MAP2K7 and PTK6 in airway of asthmatic mice and normal mice. **(C)** CeRNA network involved in regulating the expression of five prognostic genes. **(D)** Correlation between potential therapeutic drugs and corresponding targets. **(E)** Binding conformation of TBXA2R and carbacyclin (binding energy = −59.29 kcal/mol). **(F)** Binding conformation of TBXA2R and dinoprostone (binding energy = −34.08 kcal/mol). **(G)** Binding conformation of TBXA2R and fluprostenol (binding energy = −16.13 kcal/mol). **(H)** Binding conformation of TBXA2R and iloprost (binding energy = − 64.50 kcal/mol).

We analyzed ARGs and clinical data and found 61 ARGs associated with asthma control (**Figure 6A**). The LASSO Cox regression analysis was performed on ARGs, and a prediction model consisting of five ARGs (i.e., ACBD5, S100A9, CD46, MAP2K7, and PTK6) was further obtained (**Figure 6B**). The risk score for each patient was calculated as follows: Risk score = (−0.539 × ACBD5 expression level) + (0.016 × S100A9 expression level) + (−0.152 × CD46 expression level) + (0.934 × MAP2K7 expression level) + (0.182 × PTK6 expression level). Patients were divided into high- and low-risk groups on the basis of the median cutoff score. PCA based on genomic expression data showed significant distribution differences between the two groups (**Figure S1E**). We found that with increased FEV1 reversibility, the level of asthma control decreased gradually. In addition, poor asthma control was more common in the high-risk group than in the low-risk group. At the same level of FEV1 reversibility, the high-risk group was more likely to be poorly controlled than the low-risk group (**Figure 6C**). This result suggested that risk models based on ARGs could be used as a promising indicator for assessing the control status of patients with asthma. To understand the differences of immune microenvironment characteristics among different risk groups mediated by ARGs, we used ssGSEA to analyze their immune function and immune cell abundance. The high-risk group had higher T cell co-inhibition and lower degrees of immune responses, such as types I and II IFN responses, than the low-risk group, but no statistical difference was observed between the two groups (**Figure 6D**). The immune cell infiltration analysis showed that the high-risk group had a higher degree of B-cell, pDC, and Tfh infiltrations than the low-risk group (**Figure 6E**). These cells were closely related to the occurrence and development of asthma, and the difference was statistically significant (*P* < 0.05). These findings confirmed the risk-predictive value of predictive models from an immunological perspective. The results of animal experiments were the same as those of the prediction model. ACBD5 and CD46 mRNA were down-regulated in asthmatic mice, while S100A9, MAP2K7 and PTK6 mRNA were up-regulated in asthmatic mice (**Figure 7A**). There was a statistically significant difference in CD46, MAP2K7 and PTK6 (*P* < 0.05). Further immunohistochemical staining showed that compared with normal mice, CD46 was low expressed in the airway epithelium of asthmatic mice, while MAP2K7 and PTK6 were highly expressed (**Figure 7B**). The analysis of the ceRNA mechanism of the five risk genes in the model showed that S100A9, CD46, miR-328-3p, miR-20a-5p, NEAT1, MALAT1, and XIST occupied an important position and were closely associated with autophagy (**Figure 7C**). For example, miR-20a-5p, which is associated with CD46, is involved in autophagy-induced apoptosis and inflammation in asthma (43), but long-chain noncoding RNA can be used as “molecular sponge” of miR-20a-5p to participate in the regulation of target gene expression (44). Evidence showed that MALAT1 and XIST are involved in the process of autophagy of immune cells (45). Finally, eight potentially important small molecules targeting asthma were screened by the cMAP database, and seven of them had significant correlation with TBXA2R. These molecules were carbacylin, dinoprostone, fluprostenol, GR-32191, iloprost, picotamide, and U-46619, which were considered as PPAR, prostanoid, and thromboxane receptor agonists (**Figure 7D**). Carbacyclin, dinoprostone, fluprostenol, and iloprost were molecularly docked with TBXA2R to confirm their binding ability. Results showed that the affinity between them was all less than −5.0 kcal/mol. Their molecular docking patterns are shown in **Figures 7E–H**. Small molecules with high affinity might reverse or induce the biological state encoded by specific gene expression markers, thereby playing a potential therapeutic role in asthma.

## Discussion

Different types of asthma are caused by many different triggers. Th2-driven asthma is the most common and is typically characterized by elevated levels of type 2 inflammatory biomarkers, including type 2 cytokines (e.g., IL-4, IL-5, and IL-13), serum IgE, and blood eosinophils (6). However, these diagnostic markers are not specific. At present, the monoclonal antibodies approved for severe asthma have generally developed glucocorticoid resistance in patients with severe asthma, and the standard ICS–LABA treatment can no longer maintain control. In addition, monoclonal antibody drugs are often targeted for IgE or type 2 cytokines and their receptors but not for patients without allergy and noneosinophil phenotypes (3). Therefore, it is naturally inclined to poorly controlled severe asthma, and its clinical diagnosis and treatment are faced with severe challenges. Patients with severe asthma tendency should be identified early, and their evolution from mild and moderate to severe should be prevented.

The involvement of autophagy in the evolution of severe asthma has been widely explored. Studies focused on various immune cells involved in the process of asthma. Clinical observations show that NLRP3-mediated inflammatory response caused by circulating and pulmonary monocytes is a key driver of the pathogenesis of asthma. In the autophagy inhibition asthma model, NLRP3 induces monocyte activation. Autophagy inhibits NLRP3-induced monocyte activation in the asthma model. With the impaired autophagy level in circulating monocytes, the activation of NLRP3 inflammatory bodies increases. This process relies on the reprogramming of monocyte inflammatory response mediated by ARG transcription factor EB and is related to the severity of asthma (46). However, the specific role of autophagy in asthma is still controversial (47, 48). For example, neutrophils can increase the level of airway inflammation in severe asthma by destroying airway epithelial cells through autophagy (11), but the conditional knockout of ATG5 and lack of autophagy can induce neutrophil-infiltrated airway inflammation and AHR, further leading to hormone-resistant asthma (13). The difference in the results may be related to the type of immune cells and the function of specific ARGs. On the basis of the transcriptome data of patients with asthma, this study found that most of the high expression of ARGs is related to the good control of asthma. The key clinical indicators and functional enrichment analysis of patients with low-autophagy subtypes based on ARG typing point to the differences in dose and metabolism of glucocorticoids. Similar to the conclusions of previous studies (49), the present study proved that the ARG-based typing method is important in guiding the use of hormones in patients with potentially severe asthma. Future studies can focus on exploring the relationship between autophagy and glucocorticoid metabolism, and develop gene diagnosis models specially used to guide patients with severe asthma to choose glucocorticoids or monoclonal antibodies.

Gene markers are widely used in modern clinical diagnosis. In this study, SVM-RFE and LASSO logistic regression method found that SERPINB10 in ARGs is a diagnostic marker of low-autophagy subtypes. Studies showed that increased levels of SERPINB10 mRNA in epithelial cells of patients with asthma can lead to allergic airway inflammation, which is positively correlated with AHR, sputum eosinophil percentage, and exhaled nitric oxide content (50). Interestingly, the stimulation of T cell receptor with anti-CD3 antibody can upregulate the expression of SERPINB10 in Th2-polarized cells but has no effect on the expression of SERPINB10 in Th1-polarized cells, proving that it is a stable marker in non-Th2-type asthma (51). Severe asthma is dominated by Th1/Th17 cytokine responses and rarely has a Th2-mediated immune imbalance (52), whereas this phenomenon is consistent with our findings. A variety of immune cells associated with SERPINB10, such as neutrophils and dendritic cells, have also been shown to play an important role in asthma through autophagy. The enhancement of autophagy in neutrophils can activate the release of extracellular traps and aggravate asthma, whereas the enhancement of autophagy in dendritic cells can improve asthma induced by respiratory syncytial virus (53, 54). However, further research is needed to clarify the complex interaction between genes and immune cells.

Mild to moderate or severe asthma, poor control caused by environmental stimulation, medication differences, and activation of specific genes or pathways may run through the whole course of the patient (55). The analysis showed that the risk score model established in this study is a reliable index for predicting asthma control, and the role of five differentially expressed ARGs in asthma is partially supported by previous studies. Peripheral blood neutrophils in patients with asthma induce airway epithelial cells to produce S100A9 and further induce M1 macrophages to polarize through extracellular signal-regulated kinase pathway, which aggravates asthma (56). PTK6 is identified as a genetic susceptibility molecule for severe asthma (57). CD46 directly associates with autophagy, induces autophagy, and reduces oxidative stress-mediated apoptosis of respiratory epithelial cells in patients with asthma (58). Based on the DEGs of high-risk patients, the cMAP database identified different small molecules that may be effective in asthma, predominantly prostaglandin receptor agonists. Some studies found that prostaglandin D2 inhibits mediator release and antigen-induced bronchoconstriction in the guinea pig trachea by the activation of DP 1 receptors (59). Transgenic mice overexpressing the PGE 2 receptor EP 2 on mast cells show protective phenotype in allergic asthma models (60). Therefore, the small molecule found may play an important role in the treatment of patients with asthma.

On the basis of the successful classification of asthma by ARGs, this study systematically revealed the significance of ARGs in the occurrence, development, and prognosis of asthma. The key diagnostic markers and five prognostic-related genes can be used as powerful biomarkers for the prognosis of asthma. However, this study has some limitations. First, the number of transcriptome datasets studied is relatively small, and no validation set is used. In addition, the clinical information contained in the data set is limited. For example, some lung function test data cannot be used in this study. Finally, as a regulatory factor in the occurrence and development of asthma, the biological function and molecular mechanism of ARGs need to be confirmed by further study.

## Conclusion

In this study, we obtained ARGs associated with disease progression in patients with asthma. By evaluating the expression profiles of these genes, we distinguished the two subtypes and explored the relationship between autophagy, infiltrating immune cells, and asthma progression. An autophagy-related risk score model was constructed. This model is important in predicting the control of patients with asthma and can indicate the therapeutic targets and potential therapeutic drugs of asthma. Our findings can provide the predictions of individual course and control and promote the choice of better treatment strategies.

## Data Availability Statement

The datasets presented in this study can be found in online repositories. The names of the repository/repositories and accession number(s) can be found in the article/**Supplementary Material**.

## Ethics Statement

The animal study was reviewed and approved by Experimental animal ethics subcommittee of Academic Committee of Beijing University of traditional Chinese Medicine.

## Author Contributions

FY designed and conducted the whole research. JK and YZ applied for the GEO dataset analysis of asthma. WYL and ML carried out animal experiments and molecular biological analysis. ZL and WLL were responsible for the analysis of miRNAs associated with asthma. FY, HZ, and SC completed the data analysis and drafted the manuscript. XZ and JW revised and finalized the manuscript. All authors contributed to the article and approved the submitted version.

## Funding

This work was supported by the National Key R&D Program of China (2020YFC2003100, 2020YFC2003101), National Natural Science Foundation of China (No. 82174243, No. 81973715), General project of Beijing Natural Science Foundation (No. 7202110), Innovation Team and Talents Cultivation Program of National Administration of Traditional Chinese Medicine (No. ZYYCXTD-C-202001).

## Conflict of Interest

The authors declare that the research was conducted in the absence of any commercial or financial relationships that could be construed as a potential conflict of interest.

## Publisher’s Note

All claims expressed in this article are solely those of the authors and do not necessarily represent those of their affiliated organizations, or those of the publisher, the editors and the reviewers. Any product that may be evaluated in this article, or claim that may be made by its manufacturer, is not guaranteed or endorsed by the publisher.
