# Supplementary material for: Autophagy-Related Genes Are Involved in the Progression and Prognosis of Asthma and Regulate the Immune Microenvironment
Source: Front Immunol. 2022 May 10;13:897835. doi: 10.3389/fimmu.2022.897835 (PMC9127139; doi:10.3389/fimmu.2022.897835)
Supplement: Supplementary file 1 [file DataSheet_1.docx]

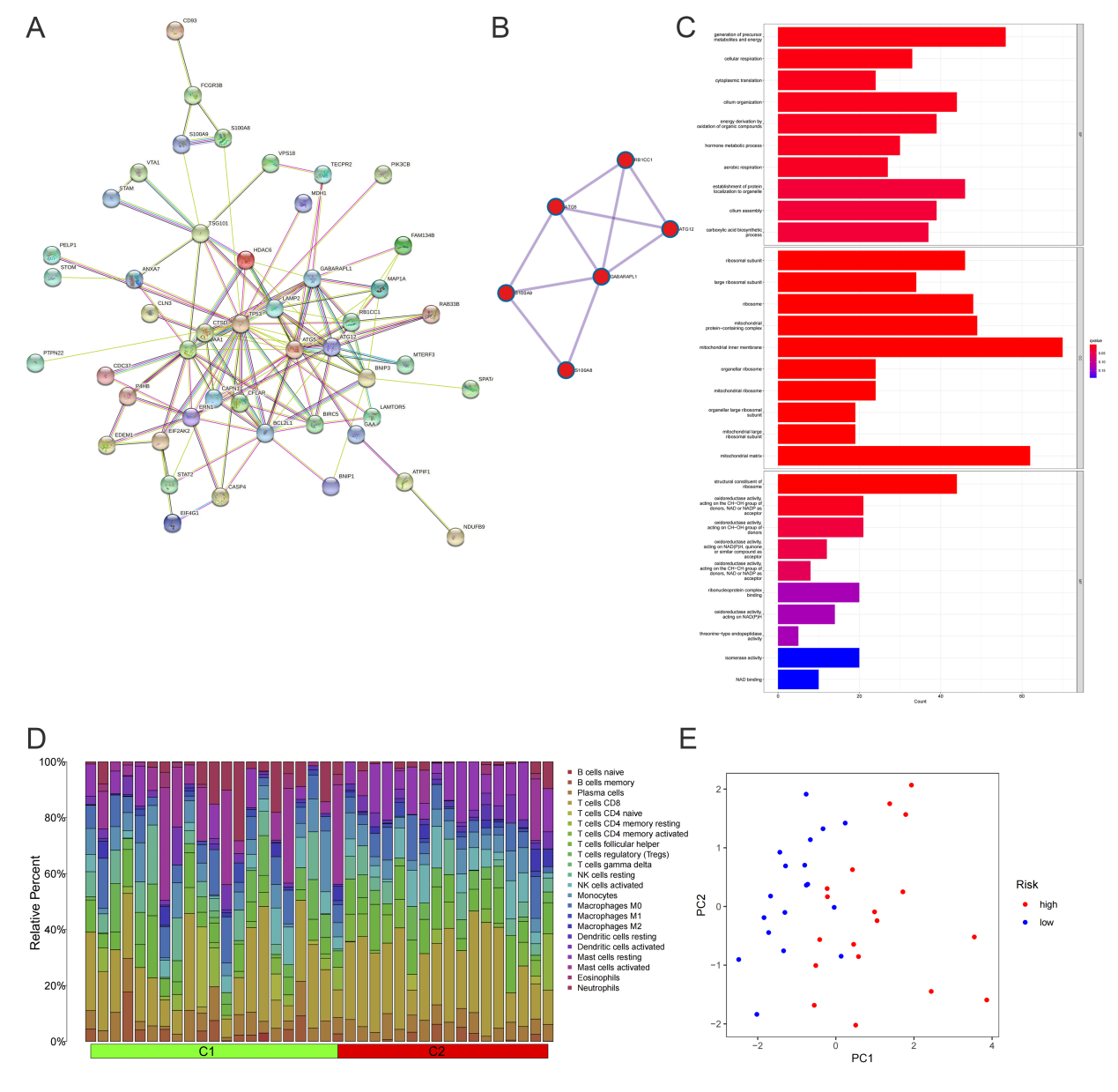


**FIGURE S1** (A) PPI networks of proteins encoded by ARGs that are differentially expressed in mild to moderate and severe asthma. (B) MCODE analysis of key modules in the PPI network. (C) GO analysis revealing the key biological processes of the C1 subtype. (D) Bar chart showing the difference and proportion of immune cell infiltration between C1 and C2 subtypes. (E) PCA of transcriptome profiles of high- and low-risk groups revealing significant differences in expression.
